# Supplementary figures and images for: A putative de novo evolved gene required for spermatid chromatin condensation in Drosophila melanogaster
Source: PLoS Genet. 2021 Sep 3;17(9):e1009787. doi: 10.1371/journal.pgen.1009787 (PMC8445463; doi:10.1371/journal.pgen.1009787)

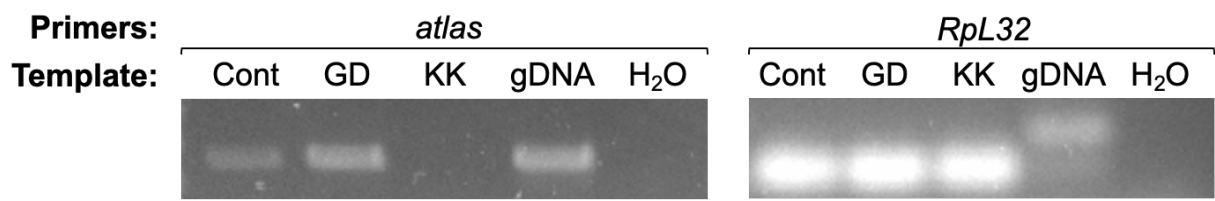

Supplement: S1 Fig — Knockdown was driven by crossing strain VDRC KK-108680 to Bam-GAL4, UAS-Dicer2. We also attempted to induce knockdown in the same manner with strain VDRC GD-17240, and we produced control flies by crossing VDRC attP strain #60100 to Bam-GAL4, UAS-Dicer2. cDNA was isolated from whole males of each strain, and a standardized amount of cDNA or control genomic DNA from w1118 was assessed for amplification of atlas and a housekeeping control gene, RpL32. The GD line did not induce detectable knockdown, but the KK line showed near-complete knockdown of atlas. Knockdown was assessed in the same way for all other RNAi lines tested; the control cross for TRIP-style RNAi lines was y v 1509 crossed to Bam-GAL4, UAS-Dicer2. (PDF) [file pgen.1009787.s001.pdf]

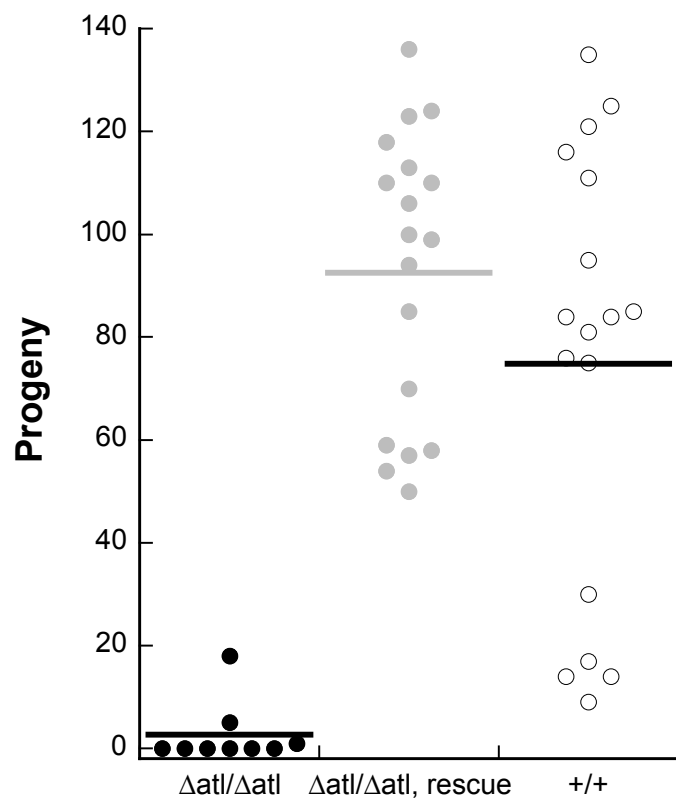

Supplement: S3 Fig — A single-pair fertility assay was used to assess fertility in males carrying a single copy of the atlas-HA genomic rescue construct in the atlas null background. These males showed no significant difference in fertility from control (w1118) males (rescue fertility: 92.6 ± 6.6; control fertility: 74.8 ± 10.3; two-sample t-test assuming unequal variances, p = 0.16). Both control and rescue males had significantly higher fertility than atlas null males (null fertility: 2.7 ± 2.0; two-sample t-tests, both p < 10−5). (PDF) [file pgen.1009787.s003.pdf]

**A**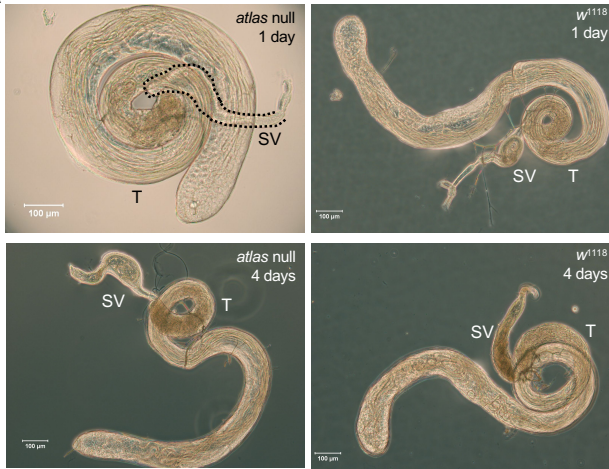**B**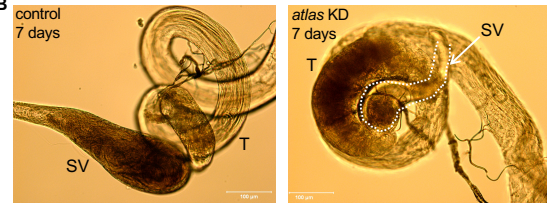**C**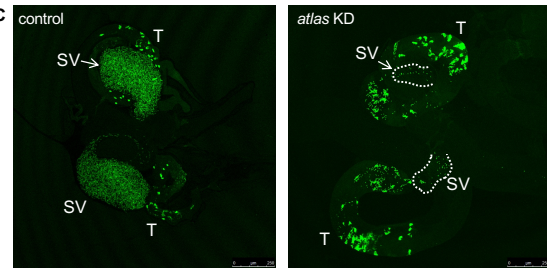

Supplement: S4 Fig — A) Phase contrast imaging of atlas null and control males ages 1 day or 4 days. Sperm accumulate in the SV by 4 days in controls, but accumulate in the basal testes of null males on days 1 and 4. The day 7 images are shown in Fig 3 of the main text. B) The same phenotype of sperm accumulation in the basal testis is observed in 7-day-old knockdown males. C) Knockdown males expressing Mst35Bb-GFP show similar patterns of spermatid nuclei to nulls, while control males accumulate many sperm nuclei in the SV (compare to Fig 3 in the main text). Control flies in B-C were generated by crossing VDRC strain #60100 (attP) to Mst35Bb-GFP; +; Bam-GAL4, UAS-Dicer2. SVs are highlighted for clarity when needed with dotted lines. (PDF) [file pgen.1009787.s004.pdf]

**A**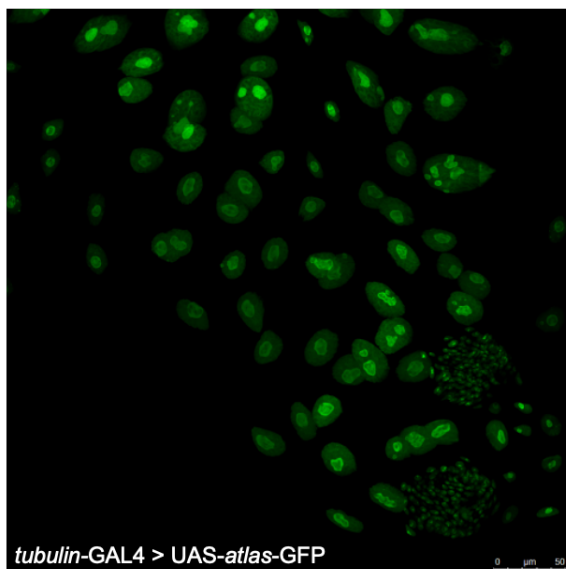**B**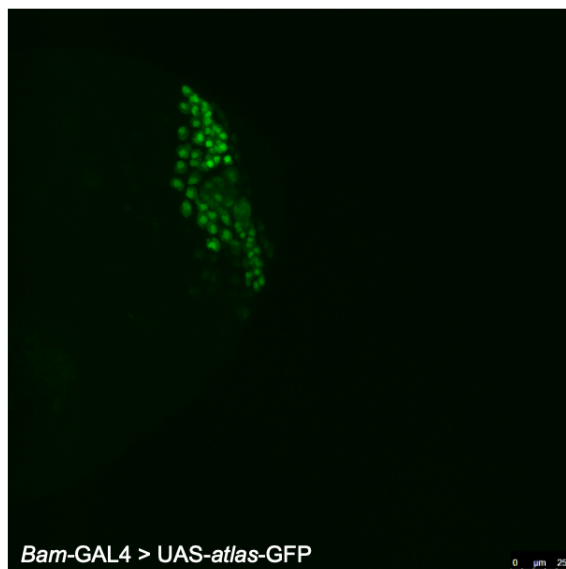

Supplement: S5 Fig — A) Dissected larval salivary glands expressing UAS-atlas-GFP under the control of tubulin-GAL4. B) Apical portion of a testis expressing UAS-atlas-GFP under the control of Bam-GAL4. In both cases, Atlas-GFP has a predominantly nuclear localization pattern. (PDF) [file pgen.1009787.s005.pdf]

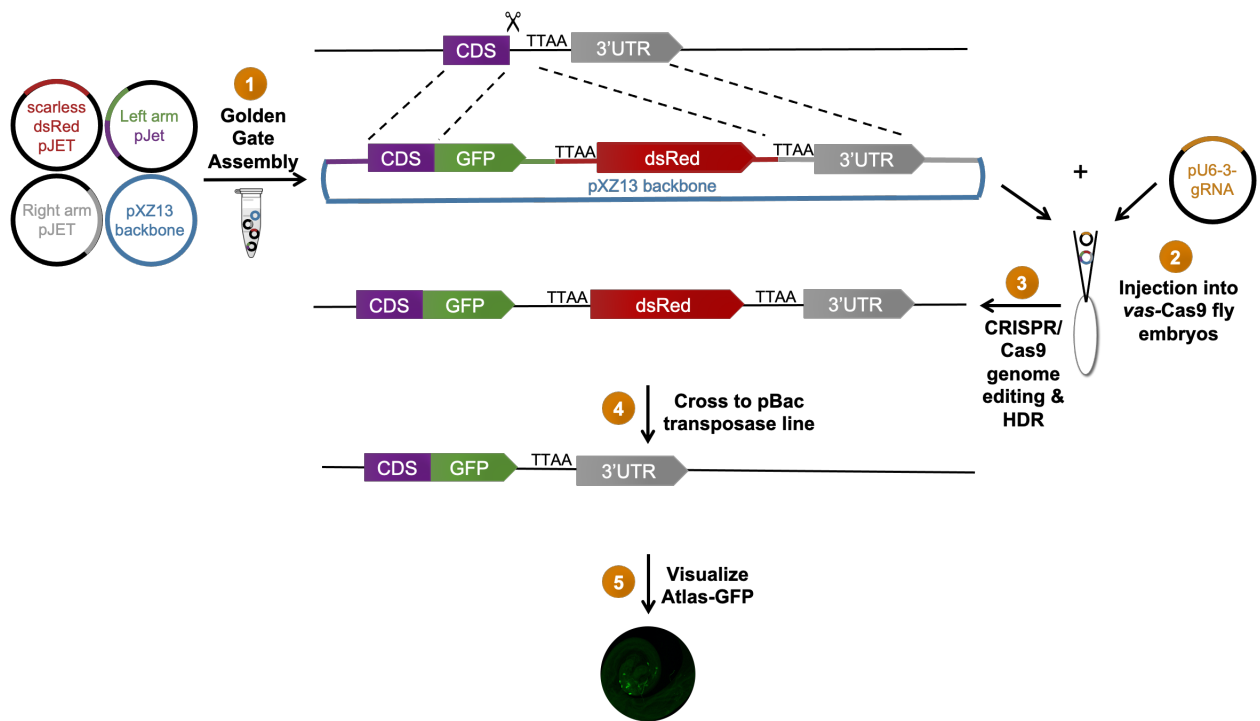

Supplement: S6 Fig — Golden Gate assembly was used to construct a plasmid carrying left and right homology arms flanking GFP placed in frame with the end of the atlas protein-coding sequence and a dsRed marker under the control of the 3xP3 promoter, which drives expression in the eye. This plasmid was injected into vasa-Cas9 flies along with a pU6.3 plasmid containing a gRNA targeting the end of the atlas coding sequence. G0 flies were crossed to w1118, and dsRed positive flies were screened molecularly for the correct atlas-GFP insert at the endogenous locus. The dsRed construct was then excised by crossing to a pBac transposase line, which removed the dsRed using flanking TTAA sequences. This protocol was adapted from Hill et al. (2019) and described at https://flycrispr.org/. (PDF) [file pgen.1009787.s006.pdf]

**A**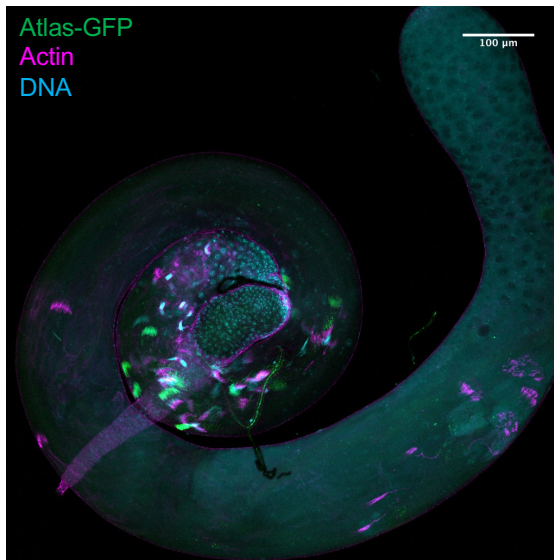**B**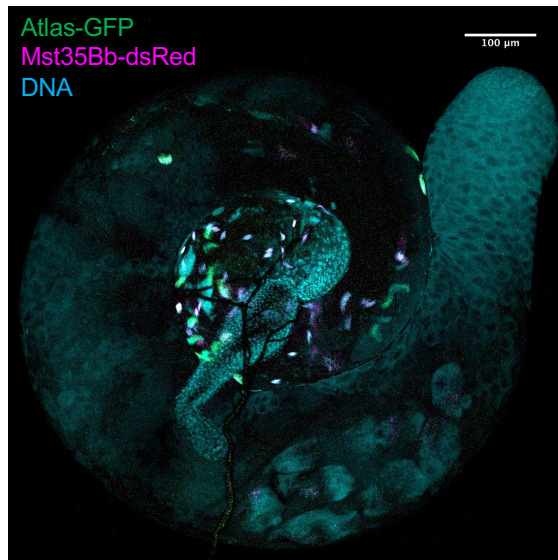

Supplement: S7 Fig — A) Whole testis dissection corresponding to Fig 5C. Small GFP-positive puncta are visible near the progressed actin cones, which may represent the removal of Atlas-GFP from condensed nuclei. Also see Fig 6D. B) Whole testis dissection corresponding to Fig 5D. (PDF) [file pgen.1009787.s007.pdf]

*Dmel*  
+

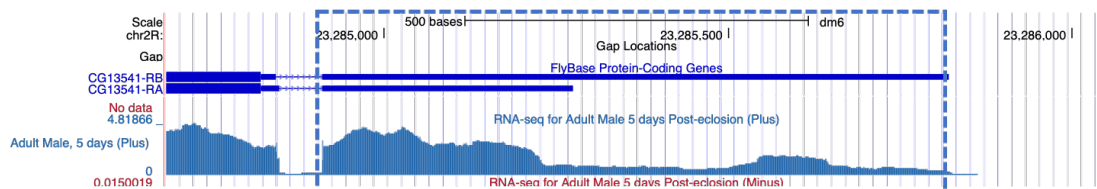

*Dsim*  
+

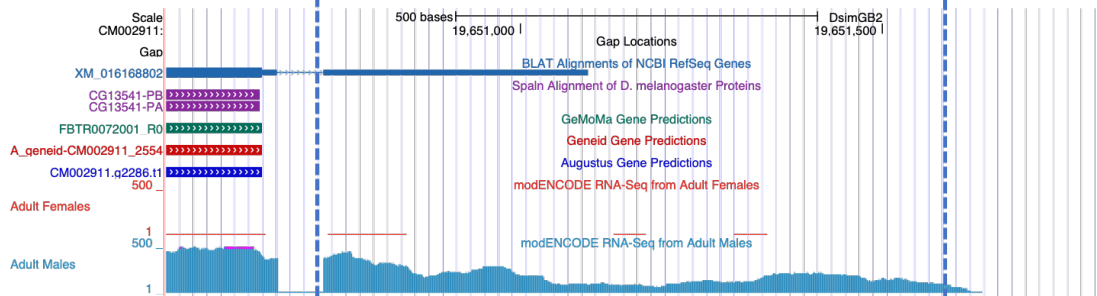

*Dsec*  
+

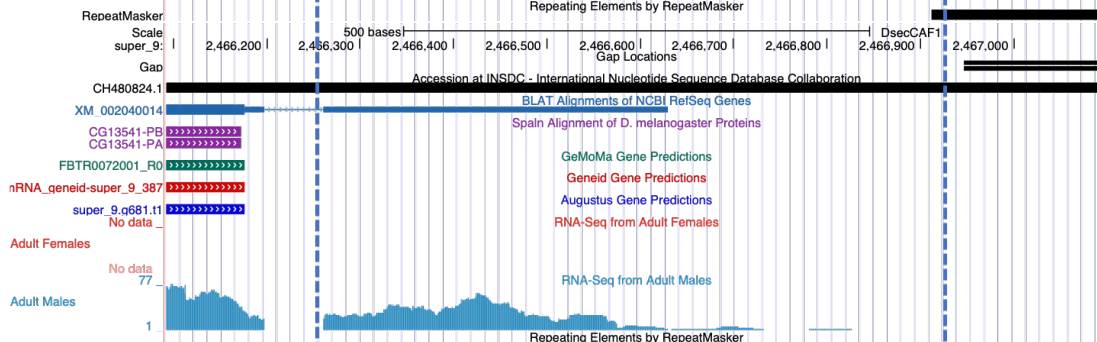

*Dmel*  
+

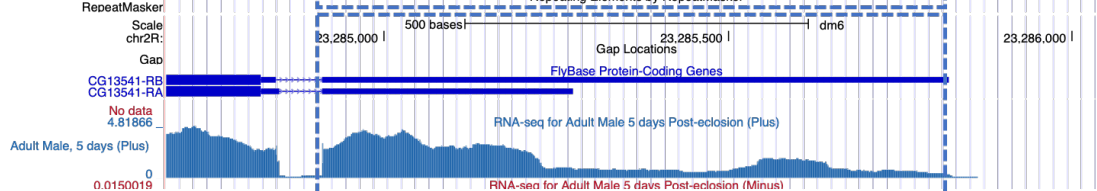

*Dyak*  
+

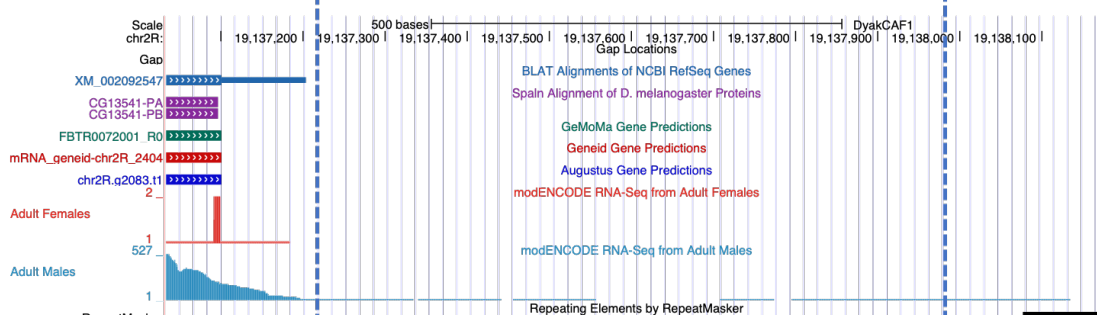

*Dere*  
+

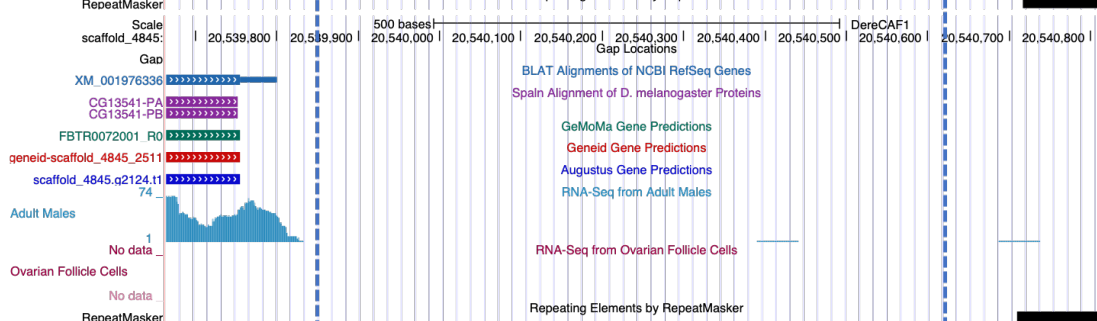

Dana

-

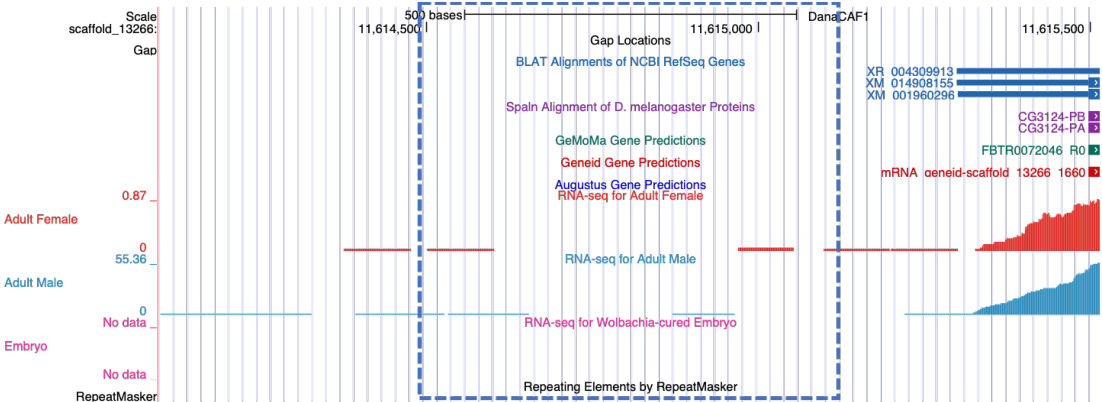

Dpse

-

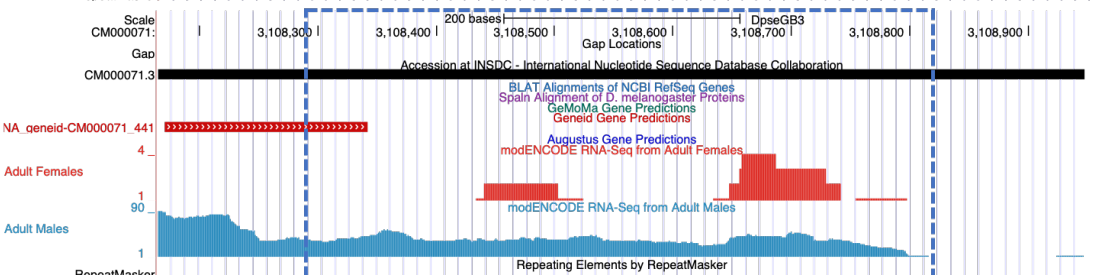

Dper

-

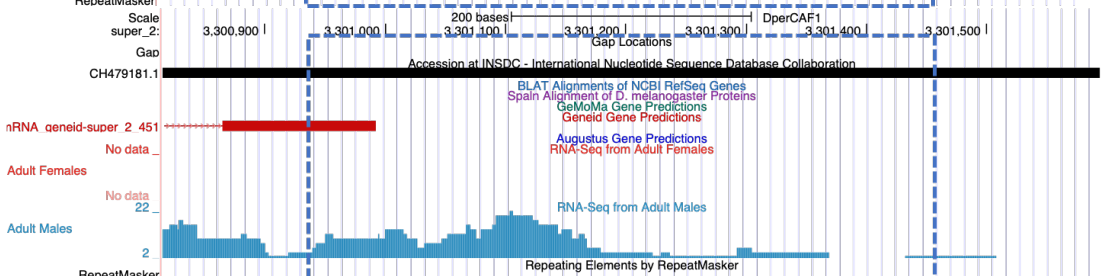

Dwil

-

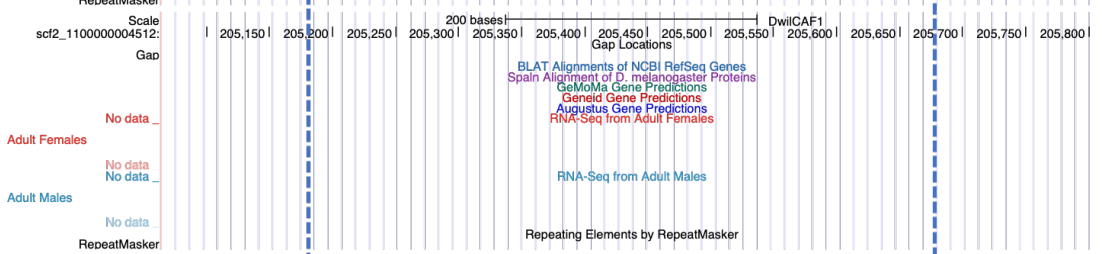

Dmoj

-

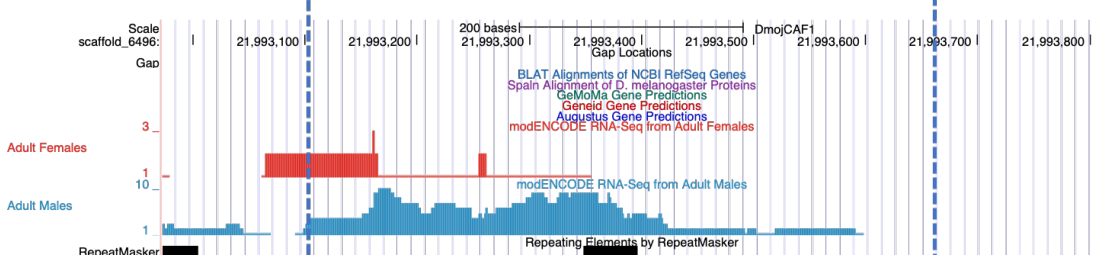

Dvir  
+

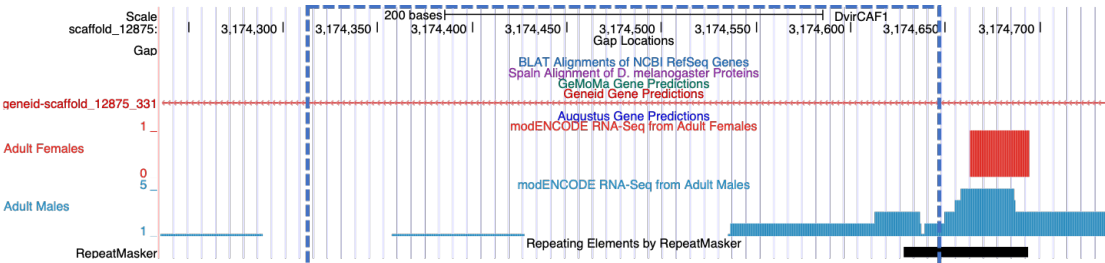

Dgri  
+

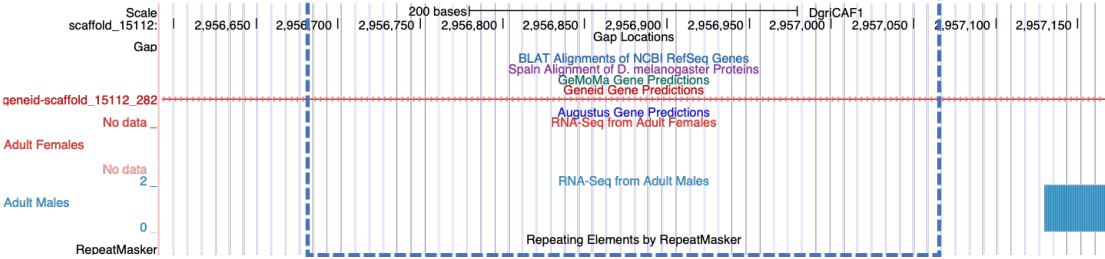

Supplement: S8 Fig — The non-coding second exon of atlas from D. melanogaster was compared with BLASTN to 11 other Drosophila species. The blue dashed line indicates the region that showed significant sequence identity in these searches. The Adult Male RNA-Seq track shows evidence of male-expressed RNA in the region. Peak heights are not comparable across species because the RNA-Seq was performed at different times. Thus, this analysis gives qualitative information about whether the conserved 3’ UTR of atlas in D. melanogaster is expressed in males in other species. The + or–symbol below each species name indicates whether the top (+) or bottom (-) strand DNA sequence matches the sequence of expressed, 3’ UTR mRNA in D. melanogaster. Expression of this region was further assessed for some species by RT-PCR; see S9 Fig. (PDF) [file pgen.1009787.s008.pdf]

A

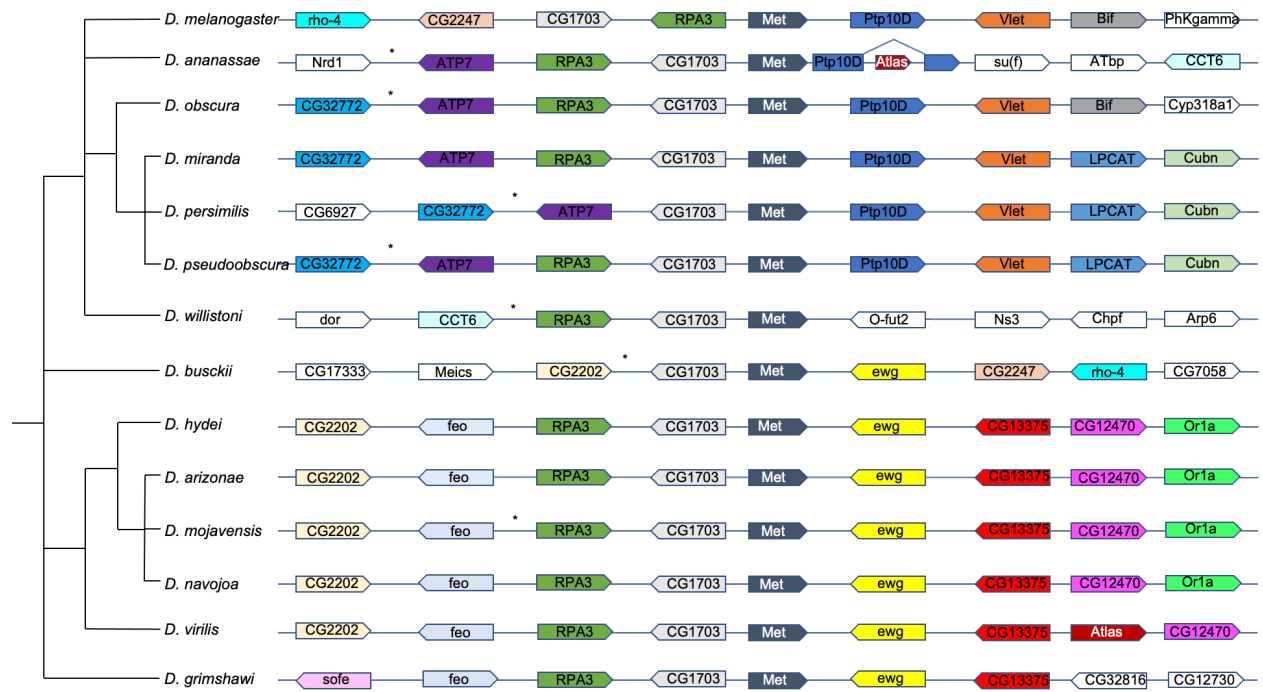

B

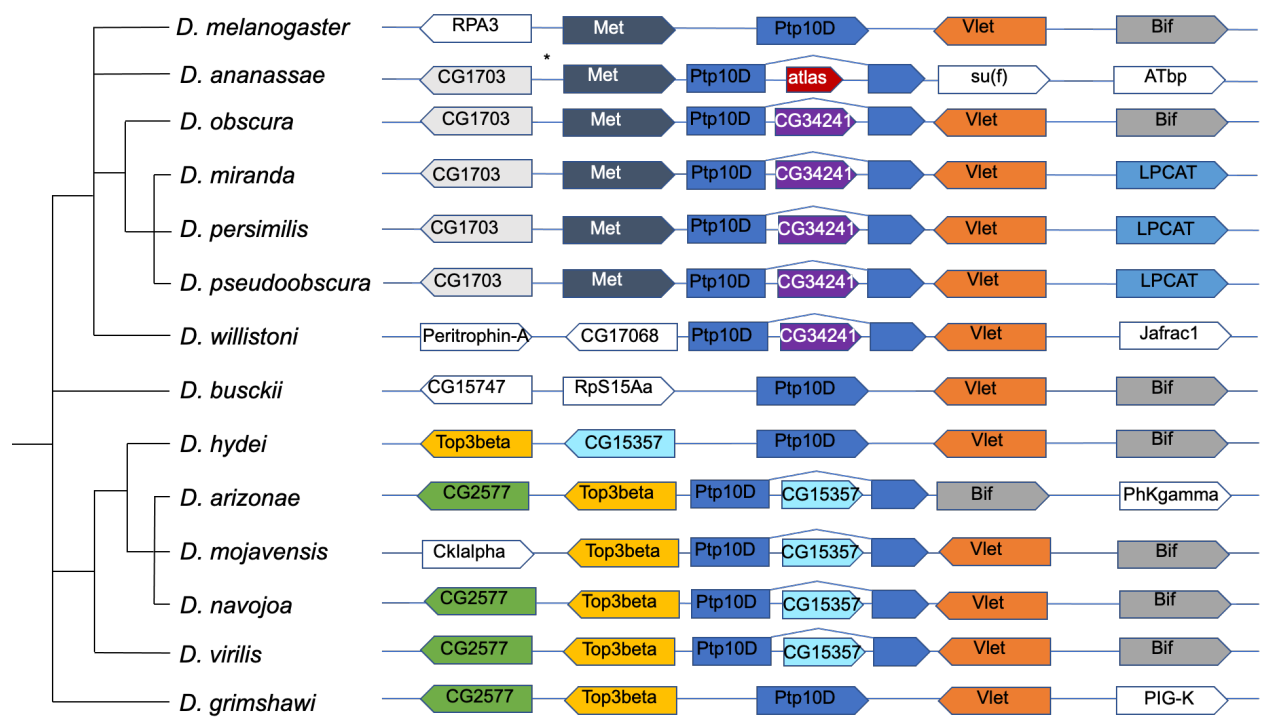

Supplement: S9 Fig — A) Atlas is found downstream of the Met gene ortholog in both D. ananassae and D. virilis. This region was therefore searched in multiple additional species. While the genomes of several species harbored unannotated genes in this general region that showed RNA-seq evidence of male expression, all such predicted genes encoded proteins that were significant BLASTP hits to D. melanogaster proteins other than Atlas. B) In D. ananassae, atlas is found in the middle of an intron of the Ptp10D gene. However, in some species, Ptp10D is no longer syntenic with Met. Therefore, we searched for atlas orthologs in and around Ptp10D across the same set of species. While orthologs of two other Drosophila gene have become inserted into a Ptp10D intron in other lineages, no additional atlas orthologs were found. In both panels, asterisks indicate unannotated genes supported by RNA-seq evidence that were confirmed with BLASTP to be homologs of genes other than atlas. (PDF) [file pgen.1009787.s009.pdf]

Atlas CDS

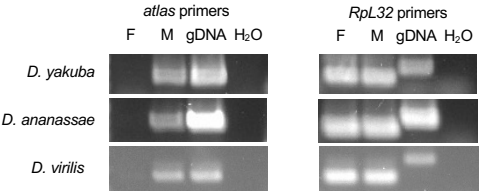

Atlas 3'UTR

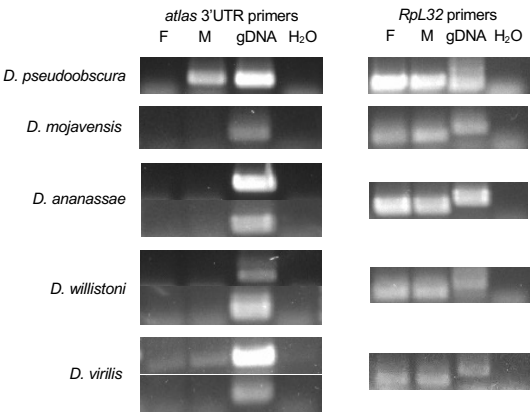

Supplement: S10 Fig — cDNA was prepared from whole males or whole females and analyzed with either atlas coding sequence primers, primers designed to a portion of the non-coding exon, or housekeeping gene RpL32 as a control. The protein-coding region is expressed in a male-specific manner in D. ananassae and D. virilis, consistent with available RNA-seq data. The non-coding region shows robust male-specific expression in D. pseudoobscura, but was not detectable in D. mojavensis (one primer pair attempted), D. ananassae (two primer pairs attempted) or D. willistoni (two primer pairs attempted). One of two primer pairs attempted gave faint, non-sex-specific amplification in D. virilis. (PDF) [file pgen.1009787.s010.pdf]
