# Supplementary material for: A putative de novo evolved gene required for spermatid chromatin condensation in Drosophila melanogaster
Source: PLoS Genet. 2021 Sep 3;17(9):e1009787. doi: 10.1371/journal.pgen.1009787 (PMC8445463; doi:10.1371/journal.pgen.1009787)
Supplement: S2 Fig — A) Graphical representation of CRISPR loss-of-function strategies. Purple boxes represent the atlas coding region, while gray represents non-coding regions of the gene. Scissors indicate locations where gRNAs targeted Cas9-mediated double-stranded breaks. The deletion allele was generated using two sgRNAs targeting either side of atlas to excise the complete coding region (CDS) and nearly all of the noncoding region. Frameshift alleles were created using one sgRNA targeting a cut at the start of the atlas coding region that was repaired with non-homologous end joining (NHEJ). B) Alignment of mutations generated in frameshift alleles. The gene’s start codon is indicated with dark green shading. Blue shading indicates bases inserted by NHEJ, orange shading indicates NHEJ deletions. All three mutations consist of net insertions or deletions that are non-multiples of three, resulting in premature stop codons indicated with underlining. The mutant alleles retain the possibility of encoding a truncated form of Atlas protein if a downstream start codon (light green shading) is used, since this codon is in-frame with the sequence encoding the protein’s C terminus. C) Predicted protein sequences encoded by the wild-type and frameshift alleles. Blue shading indicates novel amino acids created by NHEJ indel mutations. Gray shading indicates the potential truncated Atlas protein that could be encoded by the frameshift alleles if translation initiated at the downstream start codon indicated in panel B. Such hypothetical, N-terminally truncated forms of Atlas protein would contain amino acids 61–172 of the wild-type protein. Yellow shading indicates the putative nuclear localization signal. (PDF) [file pgen.1009787.s002.pdf]

A

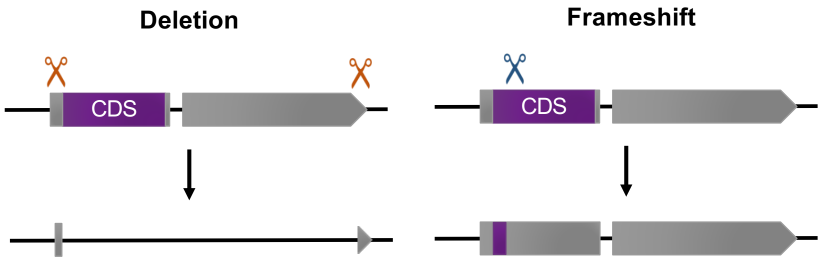

B

```
WT     GATAATAATATTATCAAAAACGTCAGAGTATTTATCGTATTGTATATTTCCAAATTACTT   60
atl52  GATAATAATATTATCAAAAACGTCAGAGTATTTATCGTATTGTATATTTCCAAATTACTT   60
atl62  GATAATAATATTATCAAAAACGTCAGAGTATTTATCGTATTGTATATTTCCAAATTACTT   60
atl86  GATAATAATATTATCAAAAACGTCAGAGTATTTATCGTATTGTATATTTCCAAATTACTT   60

WT     GTTATCACATCGAGTAAATGGGACGCAAAGGCCACAAGACTAGAA---------------  105
atl52  GTTATCACATCGAGTAAATGGGACGCAAAGGCCACAAGA-----AAAGGAAAGGCCTTCC  115
atl62  GTTATCACATCGAGTAAATGGGACGCAAAGGCCTTT------------------------   96
atl86  GTTATCACATCGAGTAAATGGGACGCAAAGGCCACAAGA-----A---------------  100

WT     ----------GGAAAGTTAAAGTCCACTCGAAGGTCCATCCTCCTCAGAAAATTAAAGCG  155
atl52  TTTTTAAGAAGGAAAGTTAAAGTCCACTCGAAGGTCCATCCTCCTCAGAAAATTAAAGCG  175
atl62  -----------GAAAGTTAAAGTCCACTCGAAGGTCCATCCTCCTCAGAAAATTAAAGCG  145
atl86  ----------GGAAAGTTAAAGTCCATTCGAAGGTCCATCCTCCTCAGAAAATTAAAGCG  150

WT     AAGCTGCCGAAAAGAGATAAACGTAGCACACTTCGTCCCAAGATTAATAATTCGAAGGTT  215
atl52  AAGCTGCCGAAAAGAGATAAACGTAGCACACTTCGTCCCAAGATTAATAATTCGAAGGTT  235
atl62  AAGCTGCCGAAAAGAGATAAACGTAGCACACTTCGTCCCAAGATTAATAATTCGAAGGTT  205
atl86  AAGCTGCCGAAAAGAGATAAACGTAGCACACTTCGTCCCAAGATTAATAATTCGAAGGTT  210

WT     TTTCACGTGAACTGCTGCAAGTACAAGATCCGTTCGAGACCCATGAGGCGCAATCAGTGC  275
atl52  TTTCACGTGAACTGCTGCAAGTACAAGATCCGTTCGAGACCCATGAGGCGCAATCAGTGC  295
atl62  TTTCACGTGAACTGCTGCAAGTACAAGATCCGTTCGAGACCCATGAGGCGCAATCAGTGC  265
atl86  TTTCACGTGAACTGCTGCAAGTACAAGATCCGTTCGAGACCCATGAGGCGCAATCAGTGC  270

WT     AACCTGGCCACCCAGTGCCCCTCCCACACGGTCGCCATCGCCCCGTCTGTGCCGCTCCTG  335
atl52  AACCTGGCCACCCAGTGCCCCTCCCACACGGTCGCCATCGCCCCGTCTGTGCCGCTCCTG  355
atl62  AACCTGGCCACCCAGTGCCCCTCCCACACGGTCGCCATCGCCCCGTCTGTGCCGCTCCTG  325
atl86  AACCTGGCCACCCAGTGCCCCTCCCACACGGTCGCCATCGCCCCGTCTGTGCCGCTCCTG  330

WT     CATCATCCTGTCCGGCGGAAGGTGGTCCATATGCCGGAAACCGTGGTCATGCACATGCGG  395
atl52  CATCATCCTGTCCGGCGGAAGGTGGTCCATATGCCGGAAACCGTGGTCATGCACATGCGG  415
atl62  CATCATCCTGTCCGGCGGAAGGTGGTCCATATGCCGGAAACCGTGGTCATGCACATGCGG  385
atl86  CATCATCCTGTCCGGCGGAAGGTGGTCCATATGCCGGAAACCGTGGTCATGCACATGCGG  390

WT     AATATGGAGGACGCCCGCCGAGTGGCGGTTAAGCACAGAAGAACCCTTATTCAAACCCAT  455
atl52  AATATGGAGGACGCCCGCCGAGTGGCGGTTAAGCACAGAAGAACCCTTATTCAAACCCAT  475
atl62  AATATGGAGGACGCCCGCCGAGTGGCGGTTAAGCACAGAAGAACCCTTATTCAAACCCAT  445
atl86  AATATGGAGGACGCCCGCCGAGTGGCGGTTAAGCACAGAAGAACCCTTATTCAAACCCAT  450

WT     GTTCCTTCGCCGGATGTGGATGATGACGAAGATACACACCAAATCGATCGGAGGTCAAGG  515
atl52  GTTCCTTCGCCGGATGTGGATGATGACGAAGATACACACCAAATCGATCGGAGGTCAAGG  535
atl62  GTTCCTTCGCCGGATGTGGATGATGACGAAGATACACACCAAATCGATCGGAGGTCAAGG  505
atl86  GTTCCTTCGCCGGATGTGGATGATGACGAAGATACACACCAAATCGATCGGAGGTCAAGG  510

WT     CAAGCCTATAGGATGGGTCAAATGAGACCCTTATCCAGCGACGTGGATGTGGATGACATG  575
atl52  CAAGCCTATAGGATGGGTCAAATGAGACCCTTATCCAGCGACGTGGATGTGGATGACATG  595
atl62  CAAGCCTATAGGATGGGTCAAATGAGACCCTTATCCAGCGACGTGGATGTGGATGACATG  565
atl86  CAAGCCTATAGGATGGGTCAAATGAGACCCTTATCCAGCGACGTGGATGTGGATGACATG  570

WT     GATGTGGACCCGTTTCAGTAG                                         596
atl52  GATGTGGACCCGTTTCAGTAG                                         616
atl62  GATGTGGACCCGTTTCAGTAG                                         586
atl86  GATGTGGACCCGTTTCAGTAG                                         591
```

C

```
WT     MGRKGHKTRRKVKVHSKVHPPQKIKAKLPKRDKRSTLRPKINNSKVFHVNCCKYKIRSRP   60
atl52  MGRKGHKKRKGLPF*---------------------------------------------   15
atl62  MGRKGL*-----------------------------------------------------    7
atl86  MGRKGHKKES*-------------------------------------------------   11

WT     MRRNQCNLATQCPSHTVAIAPSVPLLHHPVRRKVVHMPETVVMHMRNMEDARRVAVKHRR  120
atl52  ------------------------------------------------------------   15
atl62  ------------------------------------------------------------    7
atl86  ------------------------------------------------------------   11

WT     TLIQTHVPSPDVDDDEDTHQIDRRSRQAYRMGQMRPLSSDVDVDDMDVDPFQ          172
atl52  ----------------------------------------------------           15
atl62  ----------------------------------------------------            7
atl86  ----------------------------------------------------           11
```
