# Supplementary material for: A putative de novo evolved gene required for spermatid chromatin condensation in Drosophila melanogaster
Source: PLoS Genet. 2021 Sep 3;17(9):e1009787. doi: 10.1371/journal.pgen.1009787 (PMC8445463; doi:10.1371/journal.pgen.1009787)
Supplement: S11 Fig — (PDF) [file pgen.1009787.s011.pdf]

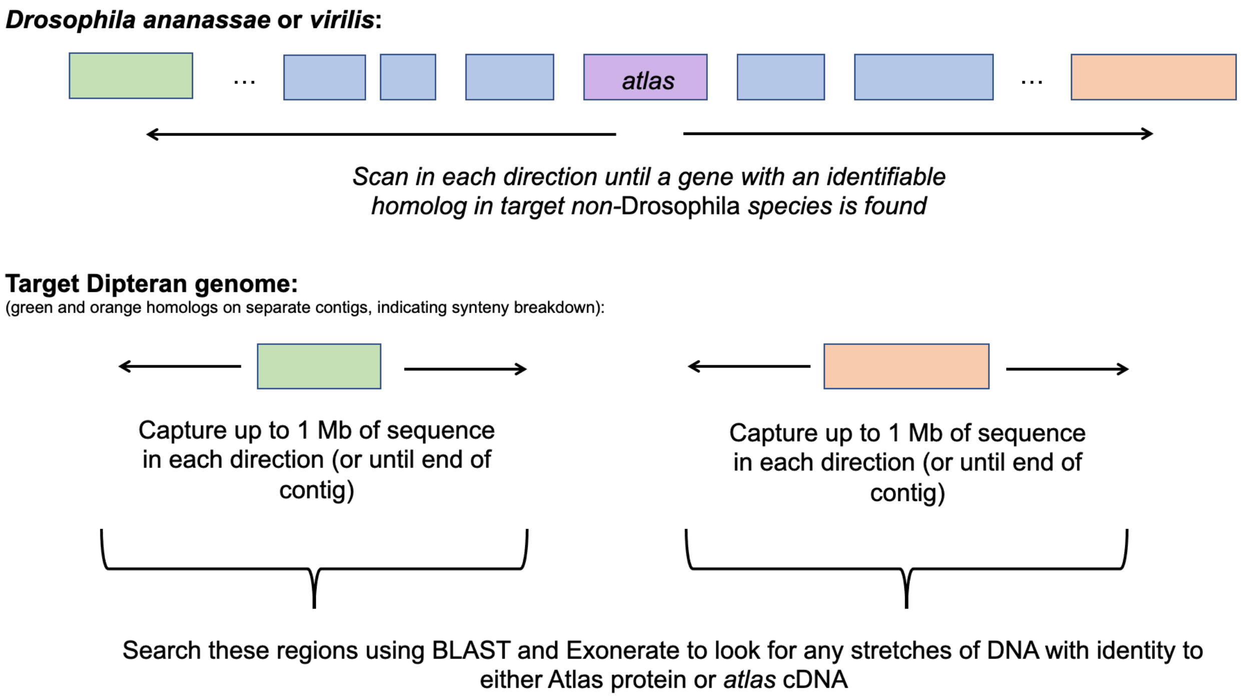

Drosophila ananassae or virilis:
…
atlas
…
Scan in each direction until a gene with an identifiable homolog in target non-Drosophila species is found
Target Dipteran genome:
(green and orange homologs on separate contigs, indicating synteny breakdown):
Capture up to 1 Mb of sequence in each direction (or until end of contig)
Capture up to 1 Mb of sequence in each direction (or until end of contig)
Search these regions using BLAST and Exonerate to look for any stretches of DNA with identity to either Atlas protein or atlas cDNA
