# Supplementary material for: A putative de novo evolved gene required for spermatid chromatin condensation in Drosophila melanogaster
Source: PLoS Genet. 2021 Sep 3;17(9):e1009787. doi: 10.1371/journal.pgen.1009787 (PMC8445463; doi:10.1371/journal.pgen.1009787)
Supplement: S1 Table — Each line shows the distribution of staged nuclear bundles dissected from one individual pair of testes. Examples of nuclear stages and the curled nuclear phenotype observed in atlas null males are shown in Fig 4. (PDF) [file pgen.1009787.s012.pdf]

Sample	Normal round nuclear bundles	Normal early nuclear bundles	Normal late canoe nuclear bundles	Normal elongated nuclear bundles	Curled elongated nuclear bundles	Normal condensed nuclear bundles	Curled condensed nuclear bundles	% Curled nuclear bundles
WT 1	0	9	10	27	0	8	0	0%
WT 2	0	6	11	13	0	9	0	0%
WT 3	0	6	7	18	0	11	0	0%
WT 4	0	7	11	20	0	9	0	0%
WT 5	0	7	8	23	0	11	0	0%
WT 6	0	7	7	10	0	2	0	0%
WT 7	0	8	15	22	0	6	0	0%
WT 8	0	8	9	6	0	0	0	0%
WT 9	0	11	11	27	0	12	0	0%
WT 10	1	11	9	19	0	11	0	0%
Null 1	0	12	13	0	17	0	8	50%
Null 2	0	6	16	0	8	0	18	54%
Null 3	0	14	12	0	6	0	3	26%
Null 4	0	12	12	0	12	0	13	51%
Null 5	0	6	23	0	9	0	24	53%
Null 6	0	2	8	0	5	0	27	76%
Null 7	0	0	0	0	3	0	23	100%
Null 8	0	5	7	0	9	0	19	70%
Null 9	0	5	7	0	10	0	17	69%
Null 10	0	6	9	0	10	0	13	60%
