# Supplementary material for: A putative de novo evolved gene required for spermatid chromatin condensation in Drosophila melanogaster
Source: PLoS Genet. 2021 Sep 3;17(9):e1009787. doi: 10.1371/journal.pgen.1009787 (PMC8445463; doi:10.1371/journal.pgen.1009787)
Supplement: S2 Table — (PDF) [file pgen.1009787.s013.pdf]

Species	Length (a.a.)	Isoelectric Point (pI)	Pairwise BLASTP bit score and e-value with Dmel	Note(s)
D. melanogaster	172	10.7	347, 3e-129	
D. simulans	172	10.6	281, 1e-103	
D. sechellia	172	10.6	293, 7e-108	
D. yakuba	178	11.4	219, 9e-79	
D. erecta	174	11.0	210, 2e-75	
D. ananassae	170	10.3	78, 2e-23	
D. bipectinata	233	10.3	62, 9e-11	Likely contains an ortholog, given significant BLAST hit and RNAseq evidence of male-specific expression, but gene boundaries are unclear. The 5' end of the ORF contains either ~100 extra codons or an intron, neither of which matches the *atlas* gene structure. Statistics reported at left are for the gene model that includes an intron, GenBank accession XP_017110044.1.
D. elegans	171	10.4	142, 2e-48	
D. ficusphila	180	10.6	147, 2e-50	
D. eugracilis	176	10.9	138, 8e-47	
D. kikkawai	No ortholog detected at mel or ana syntenic region			
D. serrata	No ortholog detected at mel or ana syntenic region			
D. rhopaloa	176	10.7	149, 4e-51	
D. biarmipes	185	10.4	158, 1e-54	
D. suzukii	179	10.6	182, 5e-64	
D. takahashii	198	10.6	165, 4e-57	
D. obscura	No ortholog detected			
D. miranda	No ortholog detected			
D. persimilis	>198	~10.3	n.s.	Found in syntenic region to *D. virilis* ortholog, but BLAST results do not support orthology; gap in *D. persimilis* assembly cuts off 3' end of predicted ORF, so length and pI values are estimates
D. pseudoobscura	232	10.1	n.s.	
D. willistoni	No ortholog detected			
D. busckii	88	5.1	34.3, 4e-8	Found in syntenic region to *D. virilis* ortholog, but low pI does not support homologous function
D. hydei	No ortholog detected			
D. arizonae	No ortholog detected			
D. mojavensis	No ortholog detected			
D. navojoa	No ortholog detected			
D. virilis	116	10.1	40, 1e-9	
D. grimshawi	No ortholog detected			
