# Supplementary material for: A putative de novo evolved gene required for spermatid chromatin condensation in Drosophila melanogaster
Source: PLoS Genet. 2021 Sep 3;17(9):e1009787. doi: 10.1371/journal.pgen.1009787 (PMC8445463; doi:10.1371/journal.pgen.1009787)
Supplement: S4 Table — (PDF) [file pgen.1009787.s015.pdf]

***atlas*-HA**

Atlas Rescue F1: GGCATGTCGACCTCGAGTACCCGGGAGCTCGAATTCTAGAcctgaaagcagaacattgta
Atlas Rescue R1: ATGGGTAAAAGATGCGGCCTCCACCGCGGTGGAGATCCATctgaaacgggtccacatcca
Atlas Rescue F2: ATGGATCTCCACCGCGGTGGAGGCCGCA
Atlas Rescue R2: AATTACCACAGTACCTACAATATATTTCCAACACACATCCtcacgtggaccggtgtccgc
Atlas Rescue F3: ggatgtgtgttggaaatatattgtaggtactgtggtaattgtttaacttctcgtttacaaaatg
Atlas Rescue F4: ATTGCCGGCGATATCGGATCCACCGGTGCCTAGGCGCGCCgcgaccgcgcacaaactcat

***atlas*-GFP**

Cloning of atlas *CDS into pENTR prior to recombination with pTWG to form atlas-GFP:*
pENTR-atl-F: caccATGGGACGCAAAGGCCACAAG
pENTR-atl-R-nostop: CTGAAACGGGTCCACATCCATG

Amplification of homology arms for Golden Gate Assembly of scarless CRISPR HDR plasmid:
Left arm F: cgtctcaggacATGGGACGCAAAGGCCACAAG
Left arm R:
cgtctcaTTAAACAATTACCACAGTACCTACAATATATTTCCAACACACATCgTCACGTGGACCGGTGCTTGTAC
Right arm F: cgtctcaagggTTAACTTCTCGTTTACAAAATGCCC
Right arm R: cgtctcagcatTTGGCGTGGGACTCATTTTGGC
